# Macrogenomic Evidence for the Origin of the Black Fly *Simulium suzukii* (Diptera: Simuliidae) on Okinawa Island, Japan

**DOI:** 10.1371/journal.pone.0070765

**Published:** 2013-08-09

**Authors:** Peter H. Adler, Yao-Te Huang, Will K. Reeves, Sam Kyu Kim, Yasushi Otsuka, Hiroyuki Takaoka

**Affiliations:** 1 School of Agricultural, Forest and Environmental Sciences, Clemson University, Clemson, South Carolina, United States of America; 2 Department of Plant Medicine, National Chiayi University, Chiayi, Taiwan; 3 United States Air Force School of Aerospace Medicine, Wright-Patterson Air Force Base, Ohio, United States of America; 4 Department of Applied Biology, Kangwon National University, Chuncheon, Republic of Korea; 5 Department of Infectious Disease Control, Oita University, Oita, Japan; 6 Institute of Biological Sciences, University of Malaya, Kuala Lumpur, Malaysia; Ecole Normale Supérieure de Lyon, France

## Abstract

To determine the geographic origin of the black fly *Simulium suzukii* on Okinawa Island, Japan, macrogenomic profiles derived from its polytene chromosomes were compared with those of mainland and other insular populations of *S. suzukii* and of the isomorphic *Simulium tani* species complex. The Okinawan population is a chromosomally unique cytoform, designated ‘D,’ which is essentially monomorphic and differs by about 27 fixed rearrangements from the chromosomal standard sequence for the subgenus *Simulium* and by two fixed differences from its nearest known relative, representing the type of *S. suzukii*, on the main islands of Japan. Chromosomal band sequences revealed two additional, sympatric cytoforms of *S. suzukii*, designated ‘A’ and ‘B,’ each with species status, in Korea, and a third cytoform, designated ‘C,’ on Hokkaido, Japan. A new cytoform, ‘K,’ of *S. tani* from Malaysia, representing the type of *S. tani*, is more closely related to cytoforms in Thailand, as are populations from Taiwan previously treated as *S. suzukii* but more closely aligned with *S. tani* and newly recognized as cytoform ‘L’ of the latter nominal species. Rooting of chromosomal band sequences by outgroup comparisons allowed directionality of chromosomal rearrangements to be established, enabling phylogenetic inference of cytoforms. Of 41 macrogenomic rearrangements discovered in the five new cytoforms, four provide evidence for a stepwise origin of the Okinawan population from populations characteristic of the main islands of Japan. The macrogenomic approach applied to black flies on Okinawa Island illustrates its potential utility in defining source areas for other species of flies including those that might pose medical and veterinary risks.

## Introduction

The biotas of oceanic islands typically owe their origins to dispersal of propagules across open water or land bridges [1]. A question of central interest regarding insular biotas is the source of the colonizers. Various genomic analyses, both chromosomal (macrogenomic) and molecular (microgenomic), have been used to reveal the relationships and geographical origins of island biotas, with varying levels of success [2], [3], [4], [5], [6]. Source-area resolution can be particularly challenging if the colonizing species or their nearest relatives are geographically widespread, with a large number of potential sources from which to colonize.

The Ryukyu ( = Nansei) Islands of Japan lie in an arc along the eastern edge of the East China Sea from the southern end of Japan toward the northern tip of Taiwan. The archipelago consists of about 140 small islands that fall geographically and faunistically into three groups: a northern cluster with a fauna similar to that of Japan, a central group (including Okinawa) with high endemism, and a southern group with faunal elements most similar to those of Taiwan [7]. The central and southern islands share a zoogeographical history separated by the Tokara Strait (Watase Line) from the northern Ryukyus and Japan proper [8].

The islands have a complex geological history of tectonic movements and volcanism, which has spawned conflicting paleogeographic hypotheses [9]. The larger islands originated from the continental shelf, whereas the smaller islands owe their origins to coral or volcanism [8]. Most geological models generally include establishment since the late Miocene, subsequent connections to the Asian mainland via land bridges in various combinations, but typically with a southern connection, at least twice during the past 1.6 million years, and isolation as an island chain for approximately the past 0.025 million years [10], [11]. The central islands, however, putatively have a longer history of isolation, dating from perhaps the late Miocene or early Pliocene, at which time they were narrowly separated from the nearest land by the deep-sea Tokara and Kerama Gaps [9].

The geographical origins of insular black flies (Simuliidae) are typically unknown even though these insects are well represented on oceanic islands [12], [13], [14], including the Ryukyu Islands, which have 18 species [15]. Okinawa Island, the largest (ca. 1,200 km^2^) member of the island chain, has one precinctive species and four species that are distributed widely in Japan and mainland Asia [16], [17], [18]. One of these species, *Simulium suzukii* Rubtsov, is widespread in temperate Asia [19]. Three years after *S. suzukii* was formally named, a new species, *Simulium ryukyuense*, was described as unique to the Ryukyu Islands [20], but later was synonymized with *S. suzukii* [18]. Whether or not *ryukyuense* is a legitimate synonym of *suzukii* has been tested (and supported) only morphologically [18]. *Simulium suzukii* also is known from Amamioshima Island and Tokunoshima Island [18], both in the central island group of the Ryukyus and within about 165 km north of Okinawa Island. *Simulium suzukii* and the isomorphic, chromosomally similar *S. tani* Takaoka & Davies of tropical Asia form a subgroup of more than 10 chromosomally differentiated forms in the *S. tuberosum* species group, some of which might represent distinct species [21], [22].

The polytene chromosomes of black flies provide a rich source of macrogenomic characters (e.g., inversions) for discovering and identifying taxonomic entities, testing reproductive isolation, and inferring evolutionary relationships [23], [24], [25]. Several hundred cryptic species and cytoforms, consequently, have been discovered and much of the systematics of the family now is based on chromosomal evidence [26]. The minuscule probability that independent inversions have identical breakpoints [24] makes them ideal for use in phylogenetic inference. Uniquely shared chromosomal rearrangements, relative to a standard sequence, thus allow relationships among simuliid taxa to be determined [27], [28]. Outgroup comparisons then enable evolutionary directionality of the rearrangements to be established, permitting phylogenetic inference [29]. The utility of the macrogenome in simuliid systematics might be related to the prominent role that chromosomal rearrangements probably have played in speciation of the family [30].

To gain insight into the origin(s) of *Simulium suzukii* on Okinawa Island and whether the Okinawan population is genetically distinct, we characterized its macrogenome and compared the band patterns of its polytene chromosomes with those of other populations of *S. suzukii* and of the closely related *S. tani* species complex. The wealth of chromosomal rearrangements in the *S. suzukii*/*tani* lineage [21], [22] suggests that a signature of the source population(s) might be found. In addition to using comparative populations of *S. suzukii* and *S. tani* previously characterized chromosomally (i.e., from China, Taiwan, and Thailand), we cast a wide net to include new material from Japan, Malaysia, and South Korea. We ensured a robust taxonomic framework by including material from the type localities of *S. suzukii* (Japan) and *S. tani* (Malaysia). Our analyses of stepwise rearrangements of the macrogenome provide evidence that the populations most closely related to the Okinawan population are on the main islands of Japan.

## Materials and Methods

### Ethics Statement

All collections of larvae and pupae were made on public land with access from public roads. No specific permissions were required to access sites or collect material, and the collections did not involve endangered or protected species.

### Collection and Preparation of Material

**Figure 1:**
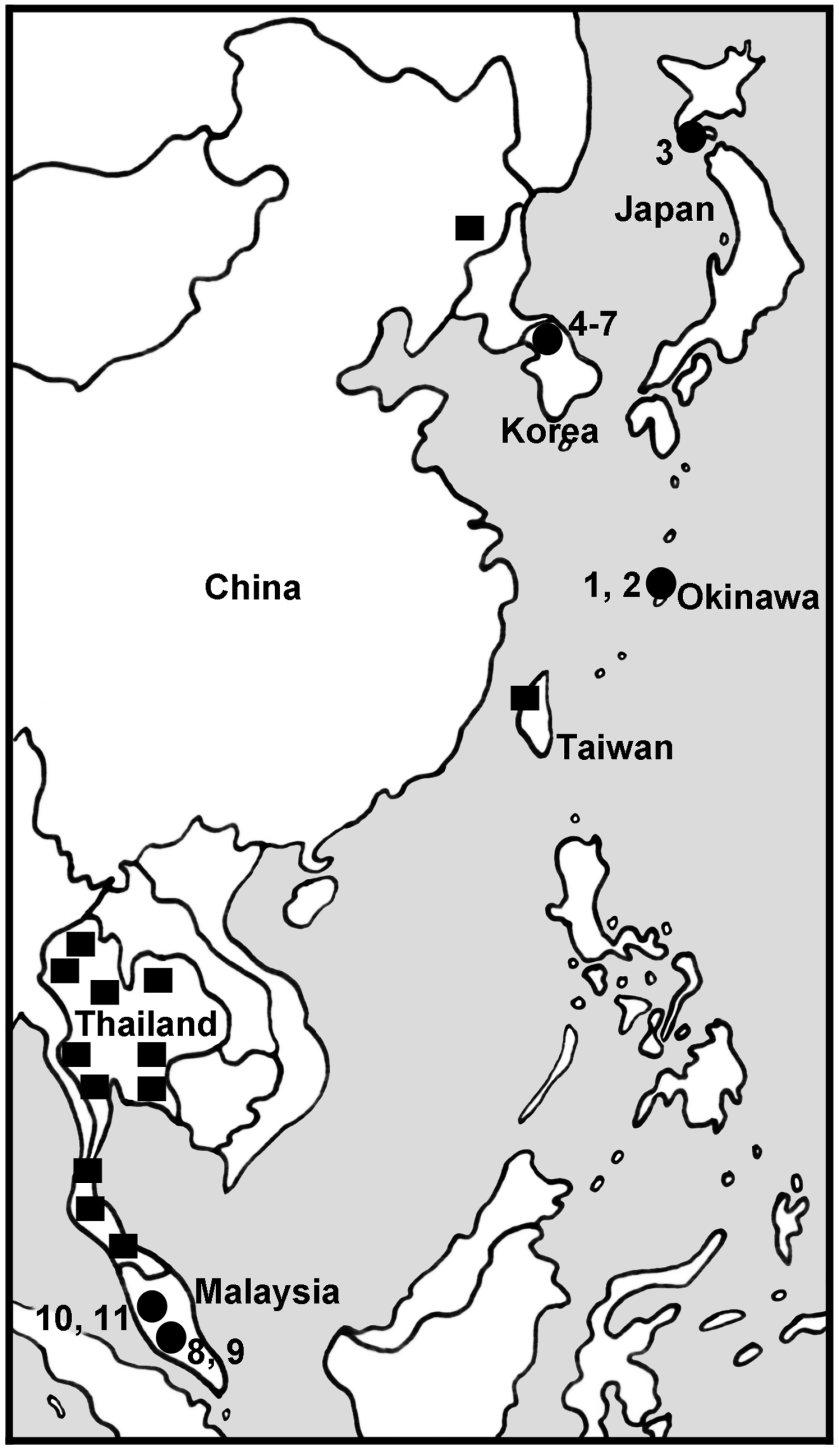
Map of chromosomally analyzed populations of *Simulium suzukii* and *Simulium tani*. Circles indicate 11 sites sampled in the current study; numbers refer to sites in Table 1. Squares indicate sites from which samples were previously analyzed [21], [22].

Larvae and pupae were collected, primarily from trailing vegetation and aquatic macrophytes, at 11 sites in 3 countries (Table 1; Fig. 1) and fixed in three changes of 1∶3 acetic ethanol. Larvae of suitable size (i.e., penultimate and ultimate instars) were Feulgen-stained for chromosomal analysis and gender determination [31]. Larval carcasses and pupae were transferred to 80% ethanol and deposited, with photographic negatives of chromosomes, in the Clemson University Arthropod Collection, South Carolina, USA.

**Table 1:** Sampling sites for larvae of *Simulium suzukii* and *Simulium tani*, with numbers of each cytoform analyzed.

Site	Location	Latitude Longitude	Elevation (m)	Date					
					Cytoform (females:males)				
					A	B	C	D	K
1	JAPAN, Okinawa Island, below Hiji Falls	26°42′40′′N 128°11′06′′E	120	8 Sep 2010				0∶4	
2	JAPAN, Okinawa Island, ca. 2.3 km downstream from Hiji Falls	26°43′44′′N 128°10′24′′E	20	14 Nov 2010				21∶19	
3	JAPAN, Hokkaido, Kaminokuni, Amano River	41°44′42′′N 140°15′17′′E	50	24 June 2012			34∶16		
4	KOREA, Gangwon-do, Hongcheon-gun, Bukbang-myeon, Wonso-ri	37°42′08′′N 127°43′30′′E	130	10 June 2010	2∶6				
5^a^	KOREA, Gangwon-do, Chuncheon-si, Namsan-myeon, Bangha-ri	37°47′19′′N 127°32′42′′E	100	20 June 2010	3∶4	7∶11			
6^a^	KOREA, Gyeonggi-do, Gapyeong-gun, Buk-myeon, Hwaak-ri	37°57′06′′N 127°34′41′′E	210	22 June 2010		10∶5			
7	KOREA, Gangwon-do, Hongcheon-gun, Ducheon-myeon, Cheonhyun-ri, Pyeongcheon	37°50′33′′N 128°00′20′′E	230	20 Aug 2011	1∶1	10∶8			
8	MALAYSIA, Selangor, Hulu Langat, Gabai Falls, 20 km E Kuala Lumpur	03°09′57′′N 101°54′29′′E	149	4 Jan 2011					6∶5
9	MALAYSIA, Pahang, Janda Baik, Sungai Lurau	03°18′13′′N 101°52′30′′E	530	22 Feb 2011					17∶11
10	MALAYSIA, Pahang, Tanah Rata, stream in front of Forest Department	04°28′44′′N 101°22′58′′E	1470	28 Jan 2011					5∶4
11^a^	MALAYSIA, Perak, stream crossing road from Iskandar Falls to Tanah Rata, after Iskandar Falls	04°20′27′′N 101°20′01′′E	580	29 Jan 2011					4∶2

a Chromosomal band patterns of 4 additional female larvae could not be read entirely (2 from Site 5, 1 from Site 6, and 1 from Site 11), and were excluded from the table and analyses; otherwise, the band sequences of all prepared larvae were read completely.

### Construction of Chromosome Maps

**Figure 2:**
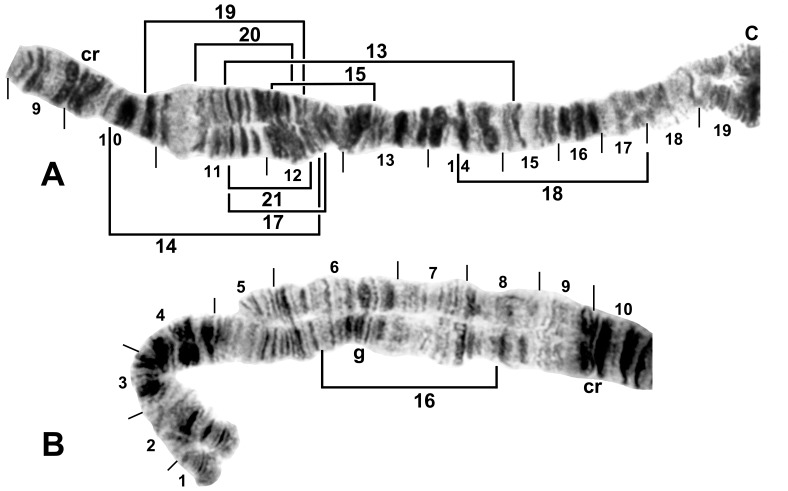
IS of male larva. C = centromere, cr = crack marker, g = glazed marker. Breakpoints of autosomal inversions IS-13–IS-21 are indicated by brackets. A. *Simulium suzukii* cytoform ‘B’ from Gangwon-do, Chuncheon-si, Korea, 20 June 2010. B. *Simulium tani* from Daping River, Taiwan, 3 December 2008.

**Figure 3:**
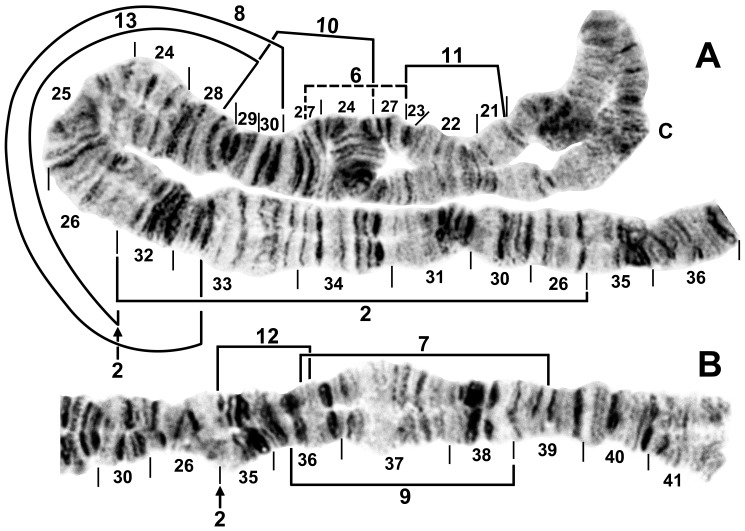
IL of male larva. Relative to the *Simulium* subgeneric standard, 3 fixed inversions (indicated only by section numbering) and IL-2 (indicated by arrows), are present. Breakpoints of autosomal inversions IL-7 to IL-13 are indicated by brackets. A. *Simulium suzukii* cytoform ‘B’ from Gangwon-do, Chuncheon-si, Korea, 20 June 2010. Y-linked IL-6 is indicated by a dashed bracket and is associated with failure to pair in the CI region. B. *Simulium tani* from Thailand.

**Figure 4:**
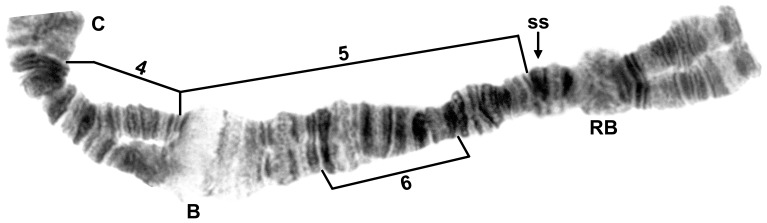
IIS of male larva of *Simulium suzukii* cytoform ‘B’. Specimen from Gyeonggi-do, Korea, 22 June 2010. B = bulge, C = centromere, RB = ring of Balbiani, ss = shoestring marker. Breakpoints of autosomal inversions IIS-4, IIS-5, and IIS-6 are shown with brackets. Inverting IIS-5 will place the bulge in standard position next to the shoestring marker. Proposed section numbers relative to the *Simulium* subgeneric standard have been reported previously [21].

**Figure 5:**
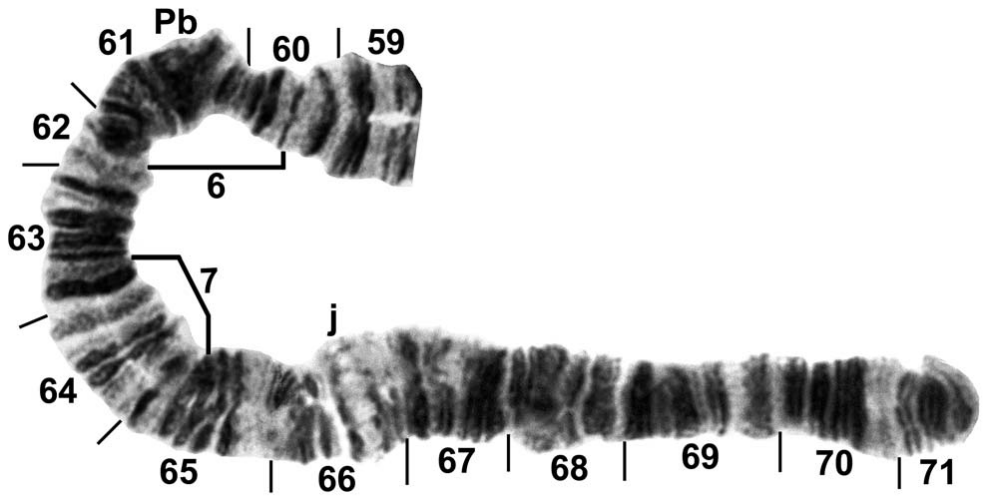
Photocomposite of distal three-quarters of IIL. Female larva (59–65) of *Simulium tani* from Janda Baik, Malaysia, 22 February 2011, and male larva (sections 65–71) of *Simulium suzukii* from Kaminokuni, Hokkaido, Japan, 22 June 2012. Breakpoints of floating inversions IIL-6 and IIL-7 are shown by brackets; j = jagged marker, Pb = parabalbiani.

**Figure 6:**
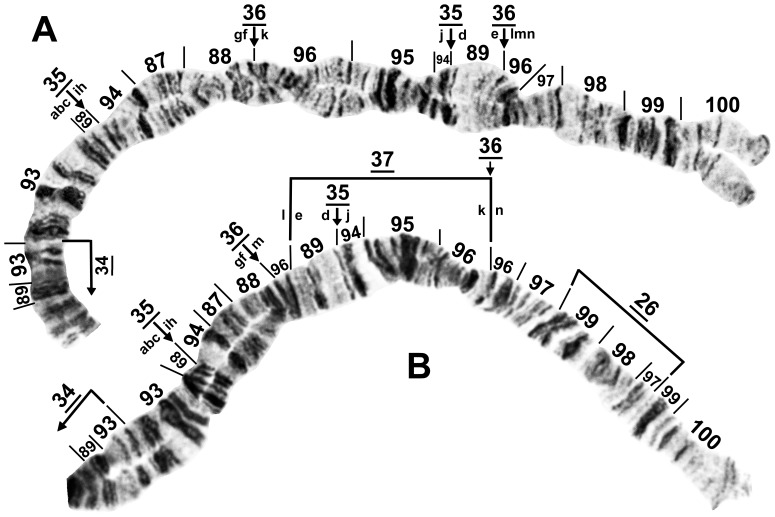
Distal half of IIIL of *Simulium suzukii*. Section numbers refer to the *Simulium* subgeneric standard. Relative to the subgeneric standard, inversions *IIIL-2* and *IIIL-3* [21] are present, plus inversions *IIIL-35*, *IIIL-36*, and (in Fig. 6B only) *IIIL-37*; sequential arrangement of the letters ‘a’ to ‘n’ produces the *Simulium suzukii/tani* standard sequence from fixed inversions *IIIL-35*, *IIIL-36*, and *IIIL-37*. A. Cytoform ‘A’ of female larva (X_0_X_0_) from Gangwon-do, Chuncheon-si, Korea, 20 June 2010. B. Photocomposite of cytoform ‘B’ of male (sections 89–93 only) and female larvae from Gyeonggi-do, Korea, 22 June 2010.

**Figure 7:**
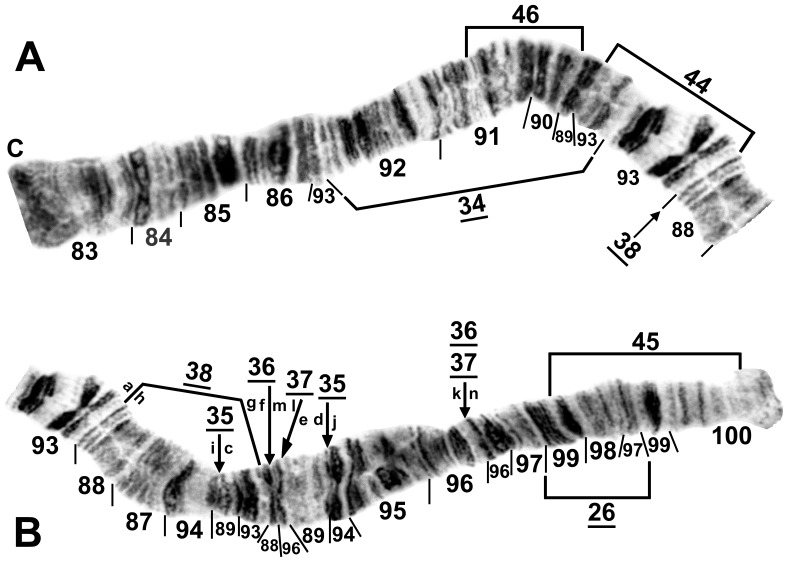
Photocomposite of IIIL of female larva of *Simulium suzukii* cytoform ‘D’. Specimen from Hiji River, Okinawa Island, 14 November 2011. Section numbers refer to the *Simulium* subgeneric standard. Relative to the subgeneric standard, inversions *IIIL-1*, *IIIL-2*, and *IIIL-3* [21], are present, plus 6 fixed inversions: *IIIL-26*, *IIIL-34*, and a complex of 4 overlapping inversions (*IIIL-35*, *IIIL-36*, *IIIL-*37, and *IIIL-38*); sequential arrangement of the letters ‘a’ to ‘n’ produces the *Simulium suzukii/tani* standard sequence from the complex of 4 inversions. Breakpoints of 3 floating inversions (IIIL-44, IIIL-45, and IIIL-46) that occur in cytoform ‘C’ are shown by brackets. A. Basal half of arm; C = centromere. B. Distal half of arm.

**Figure 8:**
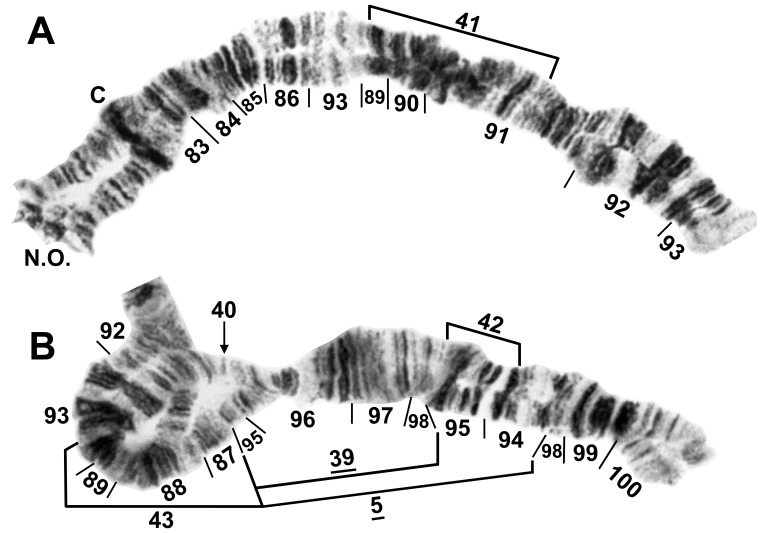
IIIL of female larva of *Simulium tani* cytoform ‘K’. Specimen from Janda Baik, Malaysia, 22 February 2011. Section numbers refer to the *Simulium* subgeneric standard. Relative to the subgeneric standard, inversions *IIIL-1*, *IIIL-2*, and *IIIL-3* [21] are present. A. Base of IIIS and IIIL showing breakpoints of autosomal inversion IIIL-41; C = centromere, N.O. = nucleolar organizer. B. Distal half of IIIL. Fixed inversions *IIIL-5* and *IIIL-39* and autosomal inversion IIIL-40 (heterozygote, arrow indicating breakpoint) are present; breakpoints of autosomal inversions IIIL-42 and IIIL-43 are shown by brackets.

**Figure 9:**
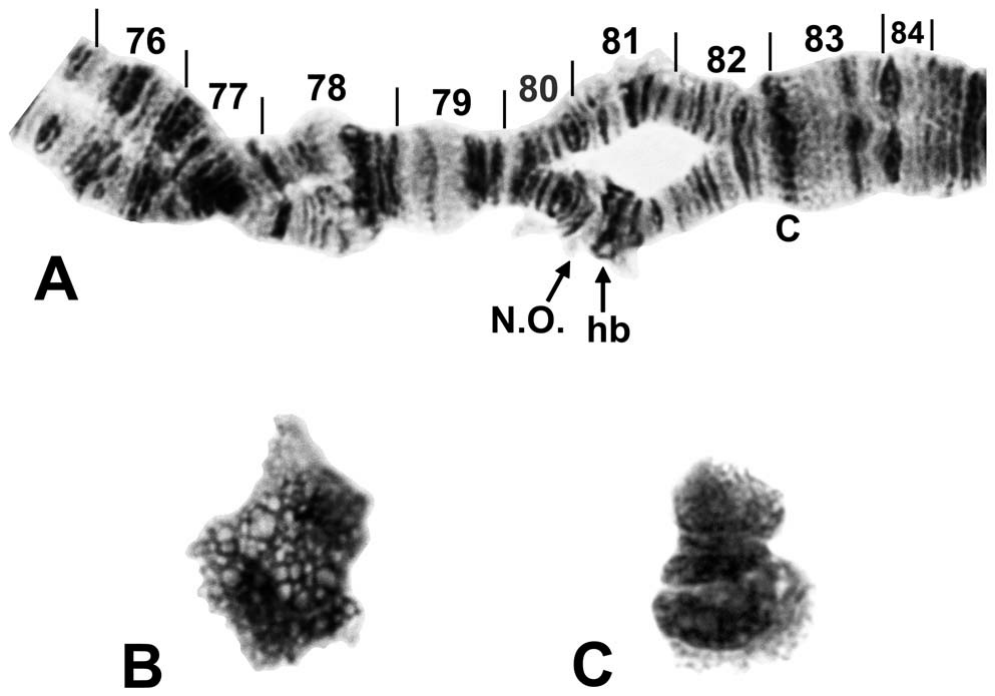
IIIS and B chromosomes of *Simulium suzukii*. A. Heterozygous band enhancement (hb) beside the nucleolar organizer (N.O.) in section 81 of IIIS in male larva of cytoform ‘D’ from Okinawa Island, 14 November 2010; C = centromere. B. B chromosome of female larva of cytoform ‘B’ from Gangwon-do, Chuncheon-si, Korea, 20 June 2010. C. B chromosome of female larva of cytoform ‘C’ from Kaminokuni, Hokkaido, Japan, 24 June 2012.

The polytene chromosomes were characterized relative to the standard maps for the subgenus *Simulium*, the *S. tuberosum* group, and the *S. tani* complex, according to procedures and terminology previously outlined [21], [22]. Chromosome arms with novel band sequences were photographed under oil immersion (1600x) on an Olympus BX40 compound microscope, and scanned into Adobe® Photoshop® Elements 8 to assemble chromosome maps. All novel chromosomal rearrangements are indicated with brackets or arrows on the maps (Figs. 2–9).

### Chromosomal Terminology and Procedures

The three chromosomes were conventionally numbered I, II, and III in order of decreasing length, each with a short (S) and long (L) arm divided by the centromere. The entire complement was divided into 100 sections of approximately equal length, following established section limits [21]. Chromosomal landmarks [25] for the subgenus *Simulium* are identified on our maps (Figs. 2–9), and all inversions are numbered. Previously recognized inversions carry the same numbers as those previously recognized for the *S. tuberosum* group [21], [22], whereas novel inversions within a chromosomal arm bear new, sequential numbers. Fixed inversions are underlined on the maps and italicized in the text; autosomal and sex-linked inversions are in standard type. For brevity, chromosomes of each population are characterized relative to the central sequence of the *S. tani* complex [21]; thus, inversions that characterize all known members of the *S. tani* complex are not repeated in the text, although the numbering of sections on our maps reflects all differences relative to the *Simulium* subgeneric standard. Polymorphic (floating) autosomal inversions of sufficient frequency were tested with a Chi-square goodness of fit test for Hardy-Weinberg equilibrium.

We identified and characterized the sex chromosomes of populations in our study when they were microscopically discernible. Any of the three chromosomes (I, II, or III) can serve as the sex chromosome. X and Y can be microscopically undifferentiated (i.e., X_0_Y_0_), or can carry rearrangements, usually inversions, which occur heterozygously in the heterogametic sex, typically the male, giving rise to a single set of differentiated sex chromosomes (X_0_Y_1_, X_1_Y_0_, or X_1_Y_1_) or to multiple (i.e., polymorphic) sex chromosomes, each associated with a different rearrangement or set of rearrangements (e.g., X_1_X_3_, X_2_Y_1_). The populations in our study had microscopically undifferentiated or variously differentiated sex chromosomes.

### Phylogenetic Inference

A phylogeny was inferred based on uniquely shared, derived chromosomal rearrangements, primarily inversions. This procedure involved two steps: (1) resolution of all rearrangements in the *S. suzukii*/*tani* lineage relative to the *Simulium* subgeneric standard for the IS, IL, IIL, and IIIS arms [25] and the IIS and IIIL arms [32]. The subgeneric standard sequence represents the most central banding pattern from which all other band sequences in the subgenus can be resolved with the fewest number of steps. (2) The *Simulium* subgeneric standard, however, lacks directionality. We, therefore, rooted our phylogeny by resolving the subgeneric standard for the IIIL arm relative to the common sequence in *Simulium* (*Boophthora*) *erythrocephalum* and by comparing band sequences that encompass the breakpoints of inversion IS-21 in *S. suzukii*/*tani* with relevant sections of the IS arm in *S. erythrocephalum*.

## Results

Of 220 larvae prepared for study from 11 newly sampled sites in Japan, Korea, and Malaysia (Fig. 1), 216 (98.2%) were analyzed completely. All larvae had three metacentric chromosomes, the nucleolar organizer in the base of IIIS (section 81), and no chromocenter or ectopic pairing of centromeres. The larvae had a total of 41 chromosomal rearrangements (Table 2) and represented five segregates. We recognize the following new cytoforms on the basis of fixed inversions, unique sex chromosomes, and distinct autosomal polymorphism profiles, and then use these features to examine evolutionary relationships.

**Table 2:** Frequency of chromosomal homologues with rearrangements in 5 cytoforms of the *Simulium suzukii/tani* lineage.

Rearrangement	Cytoform (Site^a^)				
	*n* = number of larvae				
	A (4, 5, 7)	B (5, 6, 7)	C (3)	D (1, 2)	K (8–11)
	*n = *17	*n = *51	*n = *50	*n = *44	*n = *54
IS-13		0.01			
IS-14	0.03				
IS-15					0.04
IS-16			0.46^b^		
IS-17			0.02		
IS-18			0.01		
IS-19			0.01		
IS-20	0.03				
IS-21	0.03	0.06			
IL-2	0.79	1.00	1.00	1.00	1.00
IL-6		*^c^			
IL-7					0.02
IL-8					0.01
IL-9					0.01
IL-10			0.02		
IL-11			0.02		
IL-12			0.04		
IL-13					0.26^ d^
IL-eb		**^e^			
IIS-4			0.01		
IIS-5	0.68	0.03			
IIS-6		0.01			
IIL-6		0.01			
IIL-7					0.41^f^
IIIS-hb1		0.01			
IIIS-hb2				0.04	
IIIL-5					1.00
IIIL-26	**	1.00	1.00	1.00	
IIIL-34	1.00	1.00	1.00	1.00	
IIIL-35	1.00	1.00	1.00	1.00	
IIIL-36	1.00	1.00	1.00	1.00	
IIIL-37	*	1.00	0.27^g^	1.00	
IIIL-38			0.31^h^	1.00	
IIIL-39					*, **
IIIL-40					**
IIIL-41					**
IIIL-42					**
IIIL-43					**
IIIL-44			0.01		
IIIL-45			0.01		
IIIL-46			0.01		

a Site locations are given in Table 1 and Fig. 1. ^b^In Hardy-Weinberg equilibrium (ss = 15, si = 24, ii = 11; s = standard, i = inverted; χ^2^ = 0.06, df = 1, P>0.05). ^c^* = Linked to the Y chromosome. ^d^Not in Hardy-Weinberg equilibrium at Site 9 (ss = 16, si = 6, ii = 6; χ^2^ = 7.25, df = 1, P<0.01). ^e^** = Linked to the X chromosome. ^f^In Hardy-Weinberg equilibrium at Site 9 (ss = 12, si = 9, ii = 7; χ^2^ = 3.16, df = 1, P>0.05). ^g^In Hardy-Weinberg equilibrium (ss = 23, si = 23, ii = 4; χ^2^ = 0.28, df = 1, P>0.05). ^h^In Hardy-Weinberg equilibrium (ss = 25, si = 23, ii = 2; χ^2^ = 1.39, df = 1, P>0.05).

### Korean Populations: Cytoforms ‘A’ and ‘B’

The band sequences of larvae from Korea represented two cytoforms, here designated ‘A’ (17 larvae) and ‘B’ (51 larvae).

Cytoform ‘A’ carried *IIIL-34*, *IIIL-35*, and *IIIL-36* (Fig. 6A) as fixed inversions and IL-2 (Fig. 3) and IIS-5 (Fig. 4) as common floating inversions. IIIL was the sex-chromosome arm. The differentiated Y_1_ chromosome of ‘A’ was characterized by IIIL-37 and paired with one of two X chromosomes, of which X_1_, but not X_0_, carried IIIL-26. Sex chromosomes, with numbers of individuals, combined as follows: X_0_Y_1_ = 8, X_1_Y_1_ = 3, X_0_X_0_ = 5, X_1_X_1_ = 0, and X_0_X_1_ = 1. Three infrequent floating inversions occurred in IS (Table 2; Fig. 2A). The mean number of heterozygous autosomal inversions per larva was 1.06. One female larva had an amorphously banded, vacuolated, heterochromatic supernumerary (B) chromosome. One male larva of ‘A’ from Site 5 had a mermithid nematode in its hemocoel.

Cytoform ‘B’ was fixed for *IL-2* (Fig. 3), *IIIL-26*, *IIIL-34*, *IIIL-35*, *IIIL-36*, and *IIIL-37* (Fig. 6B), and had X and Y sequences in the base of IL, continuing through the centromere region. All male larvae of ‘B’ carried IL-6 heterozygously, with unpairing of the centromere region (Fig. 3A), whereas all females were homozygous standard for IL-6, with the centromere region either paired or unpaired, indicating two separate X chromosomes, one being microscopically undifferentiated (X_0_, frequency = 0.68), and the other (X_1_, frequency = 0.32) with slight elaboration of bands (IL-eb) on either side of the centromere band. Unlike in cytoform ‘A,’ IIS-5 was virtually absent (Table 2). Four additional low-frequency inversions (IS-13, IS-21, IIS-6, and IIL-6) were found (Table 2, Figs. 2A, 4, 5) One male larva had a large heterochromatic block (IIIS-hb1) in section 81 immediately before the nucleolar organizer of one homologue. The mean number of heterozygous autosomal inversions per larva was 0.16. A female larva had the same form of B chromosome (Fig. 9B) as in cytoform ‘A.’ Two female and two male larvae of ‘B’ from Site 5 were infected with mermithid nematodes.

The sympatric occurrence of ‘A’ and ‘B,’ including collections of larvae of both cytoforms from the same two streams (Sites 5 and 7), allowed us to evaluate the possibility of reproductive isolation. Fixation of IL-2 in ‘B’ but not ‘A,’ unique sex chromosomes, and markedly different frequencies of IIS-5 indicated that the two cytoforms were reproductively isolated, whereas the uniquely shared IS-21 and IIS-5 polymorphisms suggested that ‘A’ and ‘B’ have a common ancestor.

### Hokkaido (Japan) Population: Cytoform ‘C’

Larvae from Hokkaido, representing cytoform ‘C,’ were characterized by fixed inversions *IL-2* (Fig. 3), *IIIL-26*, *IIIL-34*, *IIIL-35*, and *IIIL-36*. Three autosomal inversions (IS-16, IIIL-37, and IIIL-38) each occurred in more than one-quarter of all homologues and were all in Hardy-Weinberg equilibrium (Table 2; Figs. 2B, 7). Ten additional inversions (IS-17, IS-18, IS-19, IL-10, IL-11, IL-12, IIS-4, IIIL-44, IIIL-45, and IIIL-46; Figs. 2, 3A, 4, 7) were unique to cytoform ‘C,’ but in low frequency (Table 2). Of 29 larvae that carried either or both IIIL-37 and IIIL-38, 79.3% had both, suggesting partial linkage. Sex chromosomes were undifferentiated. The mean number of heterozygous autosomal inversions per larva was 1.74. One male and one female larva had B chromosomes (Fig. 9C). ‘C’ was the only cytoform with IIIL-37 and IIIL-38 as floating inversions.

### Okinawan Population: Cytoform ‘D’

All 44 larvae from Okinawa shared a common band sequence, representing a new cytoform, ‘D,’ characterized by fixation of *IL-2* (Fig. 3), *IIIL-26*, *IIIL-34*, and a cluster of four overlapping inversions (*IIIL-35*, *IIIL-36*, *IIIL-37*, and *IIIL-38*) in the distal half of IIIL (Fig. 7). The population was virtually monomorphic; undifferentiated sex chromosomes (X_0_Y_0_) predominated and floating inversions were absent. Two of 19 males were heterozygous for a slightly enhanced band (IIIS-hb2) in section 81 beside the nucleolar organizer (Fig. 9A). Cytoform ‘D’ was chromosomally most similar to cytoform ‘C,’ based on the uniquely shared IIIL-38 inversion.

As in all populations of all cytoforms, larval body color was linked to gender. For instance, of 15 mature Okinawan larvae (i.e., those with fully developed white or dark gill histoblasts) scored for a color-gender relationship, 5 of 6 males were brownish, whereas 8 of 9 females were dark gray. One Okinawan larva, a female, was infected with a mermithid nematode.

### Malaysian Populations: Cytoform ‘K’

Four samples of larvae from Peninsular Malaysia represented a new cytoform of *S. tani*–‘K’–characterized by fixation of *IL-2* (Fig. 3A) and *IIIL-5* (Fig. 8B) and differentiated sex chromosomes based minimally on the X-linked IIIL-39 inversion (Table 3). IIIL-39, which alone represented X_1_, formed the basis for four derivatives: X_2_ = IIIL-39+40, X_3_ = IIIL-39+40+41, X_4_ = IIIL-39+42, and X_5_ = IIIL-39+43 (Fig. 8). In males, the standard sequence (Y_0_) predominated, although a second Y chromosome (Y_1_) with IIIL-39 was equivalent to X_1_. Autosomal polymorphisms (Table 2) included IS-15 (Fig. 2), IL-7, IL-8, IL-9, IL-13 (Fig. 3), and IIL-7 (Fig. 5). IIL-7 was in Hardy-Weinberg equilibrium at Site 9, whereas IL-13 was not.

**Table 3:** Sex chromosomes of *S. tani* cytoform ‘K’ from 4 sites in Malaysia.

Site^a^	X_1_X_1_ b	X_1_X_2_	X_2_X_2_	X_1_X_3_	X_1_X_4_	X_1_X_5_	X_1_Y_0_	X_1_Y_1_	X_2_Y_0_	X_4_Y_1_
8^c^	6						3	1		
9	11	2	3	1			5	1	4	1
10^d^	4						3	1		
11	2				1	1	2			

a Sites are identified in Table 1 and Fig. 1.

b X_1_ = IIIL-39, X_2_ = IIIL-39+40, X_3_ = IIIL-39+40+41, X_4_ = IIIL-39+42, X_5_ = IIIL-39+43, Y_0_ = standard, and Y_1_ = IIIL-39.

c An additional male larva had undifferentiated sex chromosomes.

d An additional female larva was heterozygous for IIIL-39.

All four Malaysian populations shared the same fixed-banding sequence and basic sex-chromosome system; we, therefore, conservatively consider them a single cytoform (‘K’). Site 10, however, lacked the IL-13 and IIL-7 autosomal polymorphisms in all 9 larvae, which occurred in 8%–41% and 41%–75%, respectively, of homologues at the other three sites. Although the sample size was small, the absence of IL-13 and IIL-7 suggested some genetic differentiation of the population at Site 10. This site was at an elevation more than 2.5 times higher than the other sites, and the pupae had a nearly smooth frons and slender gill filaments, whereas those at Sites 8, 9, and 11 had a frons moderately covered with tubercles and slightly thicker gill filaments.

### Phylogenetic Relationships

**Figure 10:**
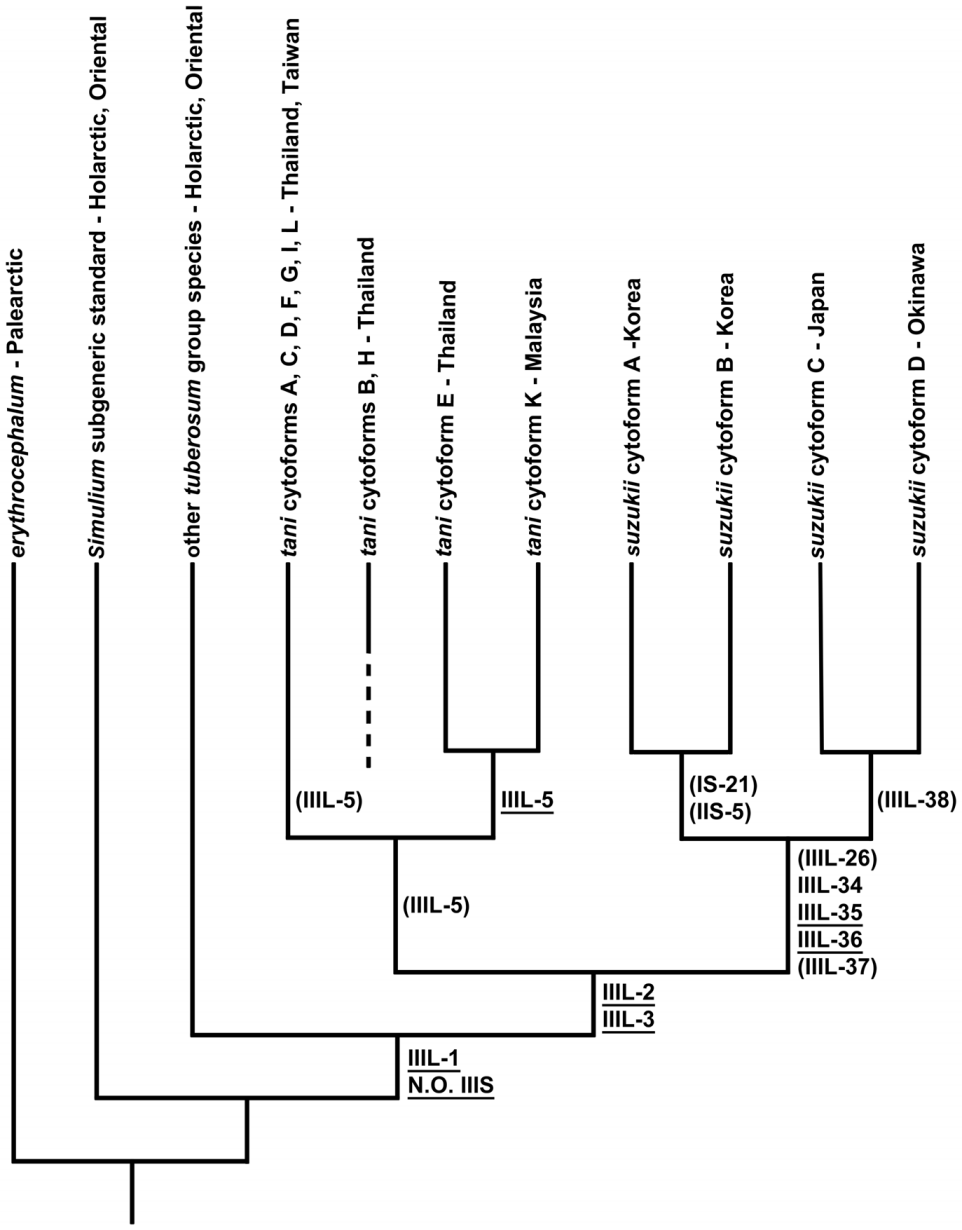
Chromosomal phylogeny of the *Simulium suzukii/tani* lineage. Uniquely shared, derived rearrangements and geographic distributions of taxa are shown. Fixed rearrangements are underlined; polymorphisms are parenthetical. N.O. = nucleolar organizer. Chromosomal relationships of cytoforms of *S. tani*, except K and L, have been reported previously [21].

The *S. suzukii* lineage is a well-defined clade differing chromosomally from the isomorphic *S. tani* lineage by at least six synapomorphic inversions (Fig. 10). Within the *S. suzukii* line, two pairs of sister taxa were recognized, including a sympatric pair of Korean cytoforms, ‘A’ and ‘B,’ united by IS-21 and IIS-5, and a pair of Japanese cytoforms, ‘C’ (Hokkaido) and ‘D’ (Okinawa), united by IIIL-38. IIS-5, which is polymorphic in *S. suzukii* ‘A’ and ‘B’ but not in ‘C’ or ‘D’ or any member of the *S. tani* complex, might represent one step in a complex of about six inversions that remove the IIS sequence of the *S. tani* complex from the subgeneric standard; when IIS-5 is present, the shoestring marker is returned to the subgeneric standard configuration (i.e., united with the bulge marker; Fig. 4). If IIS-5 eventually is confirmed as a step in the derivation of the IIS arm in the *S. suzukii/tani* line, it would then be considered present in all cytoforms, but still shared as a polymorphism in ‘A’ and ‘B.’

The stepwise derivation of four overlapping inversions (*IIIL-35*, *IIIL-36*, *IIIL-37*, and *IIIL-38*) in the Okinawan cytoform from the central (standard) sequence of the *S. suzukii/tani* lineage, through the mainland cytoforms, is shown below, where the letters ‘a’ through ‘n’ represent chromosomal fragments in Figs. 6 and 7 and brackets indicate inversions:

*38*

a **[**hi | cb **]** gf | ml | ed | jk | n *S. suzukii* cytoform ‘D’ (Okinawa)

*38*

a **[**hi | cb **]** gf | ml | ed | jk | n *S. suzukii* cytoform ‘C’

*37*

abc | ihgf **[**ml | ed | jk **]** n *S. suzukii* cytoform ‘B’

*36*

abc | ihgf **[**kj | de **]** lmn *S. suzukii* cytoform ‘A’

*35*

abc **[**ihgfed **]** jklmn hypothetical.

abcdefghijklmn *S. suzukii/tani* standard sequence.

This sequence of inversion steps (*IIIL-35*, followed sequentially by *IIIL-36*, *IIIL-37*, IIIL-38 [floating] and *IIIL-38* [fixed]) is the most parsimonious derivation relative to the outgroups–the only stepwise resolution that can be accomplished in the least number of steps and still involve intermediates represented in extant populations. The derivation suggests that a population with only *IIIL-35* exists or did at one time; alternatively *IIIL-35* and *IIIL-36* might have become fixed in the same ‘A’-like ancestor.

## Discussion

### Cryptic Species and Taxonomic Status of Cytoforms

Of the five new cytoforms, two are unequivocally distinct species. The two sympatric cytoforms ‘A’ and ‘B’ of *S. suzukii* in Korea fulfill all cytogenetic criteria [23] for reproductively isolated, albeit cryptic, species: fixed-sequence differences, unique sex chromosomes, and different autosomal polymorphism profiles. *IL-2*, *IIIL-26*, and *IIIL-37* are fixed in ‘B’ but autosomally polymorphic (IL-2), X linked (IIIL-26), or Y linked (IIIL-37) in ‘A.’ The phenomenon of a single chromosomal rearrangement assuming different roles (e.g., sex linked, fixed, autosomally polymorphic) in separate taxa is well known among simuliids [25], including members of the *S. tani* complex [21]. Although sex-chromosome polymorphisms are common within a species [26], nonhomologous sex chromosomes–III in ‘A’ and I in ‘B’–are nearly always associated with separate species [33]. Different autosomal polymorphisms and significantly different frequencies of shared inversions (e.g., IIS-5) further support separate species status. ‘A’ has a mean level of heterozygosity about 6.6 times that of ‘B.’

The type locality of *S. suzukii* is an unspecified location on the main islands of Japan. The name *S. suzukii*, therefore, applies to cytoform ‘C,’ the only cytoform known from the main Japanese islands. However, the issue of allopatry clouds the determination of whether either Korean species is conspecific with the type of *S. suzukii*. Although at least one of them will require a new name and formal description, application of a name for either would be premature without more sampling. Allopatry also presents a challenge in evaluating the conspecificity of ‘D’ on Okinawa with the type of *S. suzukii* (‘C’). The existence of inversions IIIL-37 and IIIL-38 as polymorphisms in northern Japan, while they are fixed on Okinawa, suggests the possibility that their frequency is clinal and that populations in southern Japan might be fixed, or nearly so, for these inversions, bridging the macrogenomic gap between ‘C’ and ‘D.’ Until further sampling enables this hypothesis to be tested, we conservatively support the morphologically based synonymy of *ryukyuense* with *suzukii*. The gender-related color dimorphism in larvae [31] of at least some mainland members of the *S. suzukii*/*tani* line carries over to Okinawa, suggesting similar selection pressures on mainland and insular populations.

The type specimen of *S. tani* is probably cytoform ‘K;’ one of our samples (Site 9) was collected within 40 km of the type locality (Fraser’s Hill, Malaysia). We anticipated that the Malaysian populations would conform chromosomally to either *S. tani* cytoform ‘B’ or ‘D’ [21], the nearest geographically studied populations, about 500 km to the north in southern Thailand. Instead, the Malaysian populations differed from ‘B’ and ‘D’ by fixation of *IIIL-5* and novel sex chromosomes. Malaysian populations, therefore, are more similar to cytoform ‘E,’ which inhabits an area about 1300 km to the north and is the only other known member of the *S. tani* complex that is fixed for *IIIL-5*. One male larva of ‘K’, without differentiated sex chromosomes or autosomal polymorphisms, was chromosomally identical to cytoform ‘E’ [21].

Although Taiwanese material originally was identified morphologically as *S. suzukii* [34], it is chromosomally more similar to the *S. tani* complex in Malaysia and Thailand by virtue of the shared IIIL-5 inversion [22] than it is to any analyzed population of the *S. suzukii* lineage. We, therefore, reassign the Taiwanese material to the *S. tani* complex and apply a new cytoform designation, ‘L.’ The Taiwan cytoform might represent a distinct species, differing from its closest relatives by three fixed inversions and unique sex chromosomes and autosomal polymorphisms [22]. The insular (allopatric) nature of the Taiwan cytoform, however, presents a challenge for evaluating these differences. The Taiwan cytoform is more than 2,000 km from the chromosomally most-similar analyzed mainland populations (Thailand). However, it might show affinities with populations in southeastern China, roughly 150 km across the Taiwan Strait, and therefore, eventually might be linked to a mainland entity.

The status of *S. suzukii* ‘J’ in northeastern China (as *S. tani* cytoform ‘J’ [21]) is uncertain. Although it has IIIL-26, the original interpretation that the remainder of its IIIL arm is equivalent to the sequence of the *S. tani* complex in Thailand [21] might have been overly simplistic. *IIIL-34* could have been overlooked, and the heterozygous IIIL-27 might have obscured additional rearrangements. In particular, the distal IIIL inversion complex of *S. suzukii* ‘B,’ ‘C,’ and ‘D’ carries a mimic inversion (IIIL-37) of *IIIL-36*, which excises one thin band and an adjacent fine band (that provides polarity), from the distalmost portion of section 96 and relocates them more proximally; the mimic inversion produces the superficial impression that sections 95–97 have the standard sequence.

### Phylogenetic Relationships

Chromosomal evidence suggests that two morphologically similar lineages evolved, one now represented in the Palearctic Region as the *S. suzukii* complex, defined as monophyletic by a complex of inversions in IIIL, and the other in the Oriental Region as the *S. tani* complex. Nine of the 11 members of the *S. tani* lineage are defined by the presence of inversion IIIL-5, which has assumed different roles (fixed, polymorphic, sex linked) in the various cytoforms. IIIL-5 was presumed, largely on biogeographic evidence, to have been present as a polymorphism in the ancestor of the *S. tani* line and subsequently lost in cytoforms ‘B’ and ‘H’ [21].

The band sequence of *S. suzukii* ‘A’ is about 22 fixed rearrangements removed from the *Simulium* subgeneric standard, 13 from the *S. tuberosum* group standard, and 2 from the previously defined [21] central sequence of the *S. tani* complex. Cytoform ‘B’ is an additional 3 fixed inversions (*IL-2*, *IIIL-26*, and *IIIL-37*) removed from ‘A’, and cytoform ‘D’ is removed by one more still (*IIIL-38*). ‘D,’ the most remote of the cytoforms in the entire *S. suzukii*/*tani* line, thus differs by about 27 fixed rearrangements from the *Simulium* subgeneric standard, 18 from the *S. tuberosum* group standard, and 7 (*IL-2*, *IIIL-26*, *IIIL-34*, *IIIL-35*, *IIIL-36*, *IIIL-37*, and *IIIL-38*) from the basic sequence of the *S. tani* complex. ‘D’ illustrates the potential for resolving complexly scrambled band sequences, allowing hypotheses of evolutionary relationships to be tested by following sequential rearrangements of the macrogenome deep into the simuliid phylogeny.

### Origins of the Okinawan Population

The Okinawan population is geographically closest (ca. 735 km) to the Taiwan collecting site [22], a distance only about 60 km greater than that between Okinawa and the nearest mainland (China, 28°18′N 121°38′E). Based purely on distance, Taiwan was a candidate source for the Okinawan population, either by dispersal across open water or via the putative physical connection of the Ryukyu Arc to the Chinese mainland through Taiwan as recently as 0.025 million years ago [11]. The relative proximity and historical connection of Taiwan to the Ryukyu chain probably explains the Taiwanese relationship of some faunal taxa in the archipelago, particularly in the southern islands [7], [35]. Our results, nonetheless, indicate a northern origin for Okinawan *S. suzukii*, possibly from southern Japan. Black flies are capable dispersers across open water for distances up to at least 100 km [36]. If *S. suzukii* on Okinawa can be explained by recent dispersal, it might have moved stepwise from Kyushu to Amamioshima (ca. 290 km) to Tokunoshima (ca. 35 km) to Okinawa (ca. 110 km). Populations in southern Kyushu and on Amamioshima and Tokunoshima, if cytogenetically similar or identical to the Okinawan cytoform, would provide support for this hypothesis. Alternatively, *S. suzukii* might have reached Okinawa earlier, when the Tokara Gap was narrow.

Founder effects would be expected to play a role, at least initially, in limiting genomic variation on remote oceanic islands. A tendency for chromosomal monomorphism characterizes not only *S. suzukii* on Okinawa, but also *Simulium aureohirtum* Brunetti on Guam and Okinawa [14], even though polymorphisms are common in this species on the mainland [37]. The only previous chromosomal study of a black fly on the Ryukyu Islands is that of *S.* (*Gomphostilbia*) *yaeyamaense*, which is precinctive to the Yaeyama Island group [38], about 370 km southwest of Okinawa Island. Although no relationships with other populations or speculation on geographical origins were presented, the species is female heterogametic and carries at least four autosomal polymorphisms [38]. Among additional remote-island simuliids, *S. norfolkense* on Norfolk Island about 1400 km from Australia has four polymorphic inversions, far fewer than its mainland counterpart [39]. *Simulium ruficorne* Macquart, one of the most widely distributed simuliids, has seven polymorphisms and differentiated sex chromosomes on the Cape Verde Islands, about 630 km from the African mainland [40], suggesting multiple colonizations or an adequately long insular presence for *de novo* polymorphisms to manifest.

Host-specific symbiotes, such as some mermithid nematodes of simuliids [41], offer an additional opportunity to test hypotheses of origin of their hosts. The discovery of an Okinawan larva of *S. suzukii* with a mermithid nematode in its hemocoel suggests that at some point, a parasitized adult black fly reached the island. Adult black flies bearing mermithids are typically sterilized, although they return to flowing water where the nematodes exit the host [42]. If this species of mermithid is specific to *S. suzukii*, at least two adults of *S. suzukii*, one of which bore a mermithid and presumably was sterile, must have reached Okinawa Island.

Our study likely does not capture the extent of macrogenomic differentiation of *S. suzukii* and *S. tani* on the mainland or perhaps even within the Ryukyu Archipelago. Given the extent of evolutionary differentiation of many taxa within the archipelago [35], cytogenetic differentiation of populations of *S. suzukii* on Amamioshima and Tokunoshima should be investigated.

The historical and recent invasions of mainland and island environments by flies of medical and veterinary importance, such as black flies [13], [14] and mosquitoes [43], [44], [45], underscore the need to locate source populations to aid in characterizing health risks and vector potential. Our study demonstrates that the macrogenomic approach can be used to define the source areas of insular black flies.
